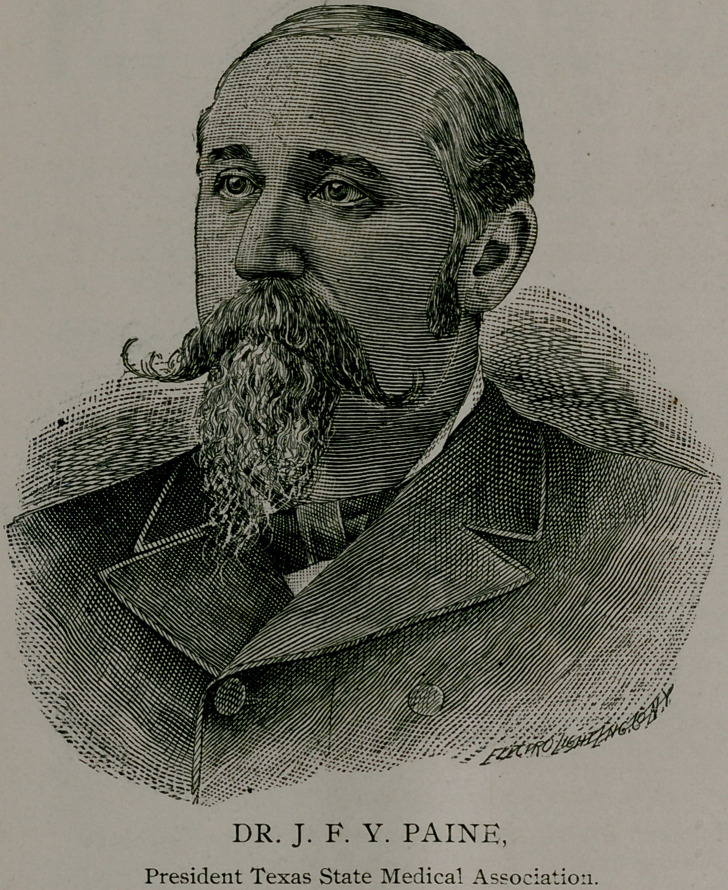# Dr. J. F. X. Paine, President-elect of the Texas State Medical Association

**Published:** 1888-05

**Authors:** 


### DR. J. F. Y. PAINE, PRESIDENT T. S. M. A.

Dr. Paine, the newly elected President of the Texas State Medical Association, is a man well fitted by character and education, to give dignity to the position, and to inspire confidence and respect. He is a gentleman of polished and dignified manners, courteous and polite, and with all a good parliamentarian. No member could have been chosen whose election would have given more general satisfaction, nor inspired more hope for the future welfare of the organization. He will make a good President, and will come near, we predict, harmonizing all conflicting elements, should any exist, now, after the very pleasant and harmonious meeting just held, under the administration of that popular and excellent man and officer, Sam. R. Burroughs. Dr. Paine is a worthy successor of a most worthy man and physician. Dr. Burroughs covered himself all over with glory, and wears his laurels with a quiet dignity quite characteristic of the man.

We present an excellent portrait of Dr. Paine, and give below a brief outline of his life. It will be seen that in a brief forty years he has crowded much of usefulness,—and it is a wish—which we know will be heartily endorsed by his hosts of friends and admirers—that he may live forty years more, of usefulness to his fellow man. It is noteworthy that Dr. Paine was not only elected unanimously, but had no opponent. His election, seemingly, was agreed upon spontaneously. Efforts have been made repeatedly to induce the noble Swearingen to accept the position of President, but in vain, and when again urged at Galveston, he declined for the third or fourth time. There is no doubt he could be elected at any meeting, if he could be inducec to accept the honor. Paine had no opponent—none wished to compete with him.

It will be seen that Dr. Paine made an early start in his career, he having been commissioned Surgeon (Major) early in 1862, in the Confederate army, when he was only 21 years of age; the youngest full Surgeon, perhaps, in either army.

Dr. Paine is a native of the Pelican State. He was born in West Feliciana Parish, La., August 16, 1840, and is of Scotch-English descent. He received an Academic education at Centenary College, Louisiana, and graduated in medicine at the University of Louisiana, in 1861, during the service of the immortal Stone. On the breaking out of the war between the States, Dr. Paine entered as a private soldier in the 20th Louisiana Regiment of Volunteers; was appointed Assistant Surgeon of the 22d Louisiana Regiment, December, 1861. After the fall of New Orleans, he served in the hospitals at Corinth and Holly Springs, Miss.; was examined May, 1862, at Columbus, Miss., by the Army Board of Medical Examiners, (Yandell, Pim and Heustis,) and, as stated, was commissioned Surgeon, with rank and pay of Major of Cavalry; assigned as Surgeon 21st Alabama Regiment, which was sent to Fort Morgan, at the mouth of Mobile bay. By seniority of commission, he took rank as Chief Surgeon of the forces constituting the defense of Mobile Bay. At the fall of these forts in '64, Dr. Paine was assigned as Chief Surgeon of General Hospital Nidelet, at Mobile, where he served till the surrender of Mobile, in '65. Hence he was ordered to Gainesville, Alabama, and took rank as Surgeon in charge of the General Hospital at that Post, and remained there till the final surrender of all the Confederate forces, in June, 1865.

Upon the declaration of peace, Dr. Paine settled in Mobile, and engaged in general practice; removed to Texas in 1874; was elect- to the Chair of Obstetrics and Diseases of Women and Children in the Texas Medical College and Hospital at Galveston, in 1875; after competitive examination, was made Dean of the Faculty in '79; was elected chairman of the Section on Gynæcology in the Texas State Medical Association, in 1885; and chairman of the Section on Practice, in 1886; was chosen Secretary to the Section on Gynæcology in the American Medical Association, in 1885; elected President Galveston County Medical Society, in same year; was one of the Vice Presidents of the Section on Public and International Hygiene of the Ninth International Medical Congress; elected to the chair of Materia Medica, Therapeutics and Hygiene, in the Medical Department of Tulane University—his *Alma Mater*, in 1885, which position he filled one term, to the entire satisfaction of the Faculty and Trustees, and with distinguished credit to himself and to Texas. Resigning this honorable position, for private reasons satisfactory to himself, he resumed practice in Galveston, where he has a large clientelle of the wealthier classes, and lives in elegance and comfort, in a beautiful home on Broadway—the Boulevard of Galveston—the fruits of his individual labors and industry. On resigning his chair in the University, at the close of the session, after repeated solicitations to reconsider his determination, he was made the recipient of a testimonial from the Faculty, in the shape of a set of resolutions, expressive of the high appreciation of his services (which were characterized as eminently satisfactory and valuable,) entertained by his colleagues, individually and collectively; and of deep and sincere regret at the necessity which induced him to sever relations so pleasant to them. These resolutions bore testimony to Dr. Paine's professional attainments and ability, no less than to those agreeable social qualities for which he is distinguished; and altogether, expressed a sincere regard for him as a teacher, a physician and a man—whom to know, is to respect; couched in language as courteous as complimentary.

Dr. Paine is an honorary member of the Alabama State Surgical and Gynæcological Association; an honorary member of the Louisiana State Pharmaceutical Association, etc. He has contributed but little to current medical literature, being kept busy by his large practice, the demands of which were such as to prevent his even being present in the hall when his election was announced amidst cheers and applause. His best papers are to be found in the Transactions of the Texas State Medical Association—notably his address as Chairman of the Section on Practice—and in the New Orleans *Medical and Surgical Journal.*

## Figures and Tables

**Figure f1:**